# *N*-Chlorotaurine, a Promising Future Candidate for Topical Therapy of Fungal Infections

**DOI:** 10.1007/s11046-017-0175-z

**Published:** 2017-07-12

**Authors:** Markus Nagl, Roland Arnitz, Michaela Lackner

**Affiliations:** 1 Division of Hygiene and Medical Microbiology, Medical University of Innsbruck, Schöpfstr. 41, 1st Floor, 6020 Innsbruck, Austria; 2 Department of Pneumology, District Hospital Vöcklabruck, Vöcklabruck, Austria

**Keywords:** Antiseptic, *N*-chlorotaurine, Inhalation, Fungi, Cystic fibrosis, COPD

## Abstract

*N*-Chlorotaurine (NCT) is a mild long-lived oxidant that can be applied to sensitive body regions as an endogenous antiseptic. Enhancement of its microbicidal activity in the presence of proteinaceous material because of transchlorination, a postantibiotic/postantifungal effect and antitoxic activity renders it interesting for treatment of fungal infections, too. This is confirmed by first case applications in skin and mucous membranes of different body sites. Recent findings of good tolerability of inhaled NCT suggest further investigations of this substance for treatment of bronchopulmonary diseases, where microorganisms play a role, particularly multi-resistant ones. The availability of a well-tolerated and effective inhaled antiseptic with anti-inflammatory properties could be a significant progress, in particular for chronic pulmonary diseases, such as chronic obstructive pulmonary disease or cystic fibrosis.

## Introduction

### Endogenous Origin of *N*-Chlorotaurine and Function in the Body

*N*-chlorotaurine (Cl–HN–CH_2_–CH_2_–SO_3_^−^, NCT), the N-chloro derivative of the amino acid taurine, is a dominant representative of long-lived oxidants produced by activated human granulocytes and monocytes [1–4], and in low concentration probably also in macrophages during the oxidative burst [1, 5–9].

Regarding the function of NCT in vivo, first a detoxification of HOCl by its reaction with taurine has been postulated [10, 11]. Second, NCT has been demonstrated to downregulate pro-inflammatory cytokines (such as tumor necrosis factor alpha, prostaglandins, nitric oxide, nuclear factor kappaB) and interleukins so that it seems to contribute to reduction of inflammation [10–17]. Third, two decades ago first data indicating antibacterial and antifungal activity were published [18–20], and killing activity of NCT against helminths was shown [21].

### Development of NCT as a Novel Antiinfective

#### Microbicidal Properties of NCT as an Antiseptic

The synthesis of the pure crystalline sodium salt of NCT (MW = 181.57 g/mol) in our laboratory succeeded [22]. A 90% stability of both the pure product and the aqueous solution at 2–4 °C per year renders it suitable for practical use [22]. Its antimicrobial properties turned out to be broad-spectrum as it is typical for active chlorine antiseptics [23]. Studies disclosed broad-spectrum bactericidal (Gram-positive and Gram-negative bacteria) [24–30], fungicidal (yeasts and molds) [27, 31–33], and virucidal activity (herpes simplex, adenoviruses, HIV, influenza so far, [34–37]) of millimolar NCT, and protozoocidal activity against amoebae, leishmaniae, and trichomonads [38–40]. Because of the unspecific oxidative mechanism of action, development of resistance is extremely improbable and was actually not detected in laboratory tests [27].

Three phenomena regarding the antimicrobial activity of NCT are meaningful, particularly after short incubation times: First, the activity of NCT against bacteria and fungi can be significantly enhanced by chlorine transfer from NCT to corresponding low molecular weight amino compounds (“transhalogenation”), above all ammonium chloride (NH_4_Cl), but also some amino acids [27, 28, 31]. In case of NH_4_Cl, the lipophilic and therefore stronger microbicidal monochloramine (NH_2_Cl) is formed [5, 27, 41]. Therefore, the activity of NCT in exudates is enhanced [27, 31].

Second, after sublethal contact times (e.g., 1 min for 1% NCT) a lag of regrowth of bacteria and fungi occurs (“postantibiotic effect”) [32, 42]. As a very important consequence, they lose virulence as proven in a mouse peritonitis model using *Staphylococcus aureus* and *Streptococcus pyogenes* [29, 42]. In yeasts, downregulation of secreted aspartyl proteinases was observed [32]. Surface chlorination of pathogens was identified as a mechanism for these findings [43].

Recently, direct inactivation of bacterial virulence factors by NCT and analogs was proven [44, 45]. For more details, see the chapter “postantifungal effect and attenuation of virulence.”

*N*-chlorotaurine has activity against biofilms, too. In a first study using 300 µM NCT, the development of *Pseudomonas aeruginosa* biofilm could be inhibited in microtitre plates in vitro [46]. In two following studies using millimolar application concentrations of NCT, bactericidal activity was found against biofilms of *S. aureus*, *Staphylococcus epidermidis*, and *P. aeruginosa* on alloy disks [47, 48].

#### Previous Clinical Studies Apart from the Bronchopulmonary System

The idea to apply NCT as an endogenous antiseptic prompted us and few other groups to perform clinical studies on the usability of the 1% aqueous solution in different body regions (for review see [49]). Tolerability was very good in the eye [50, 51], on skin [52], and mucous membranes [53, 54]. NCT was effective in external otitis [55], purulent coated crural ulcerations [52], and bacterial and viral conjunctivitis [51, 56, 57]. Tolerability in the paranasal sinuses was good, too, with some hints for improvement of symptoms in a phase IIa study [58]. In mouse models, subcutaneous NCT was well tolerated, and there were positive effects in experimental arthritis [59, 60].

## Antifungal Activities of *N*-Chlorotaurine

### Fungicidal Activity in Buffer Solution

This was tested in quantitative killing assays with different inocula. Incubation of *Aspergillus* spp., *Candida* spp., *Fusarium* spp., *Penicillium* spp., and *Alternaria* spp. in 1% NCT (55 mM) at pH 7 in phosphate buffer and at 37 °C for 1–4 h revealed a log_10_ reduction in colony-forming units (cfu) between 1 and 4 [31]. The outcome at pH 5.4 was similar. The inoculum in this first comprehensive study contained 90% hyphae and 10% conidia for molds and 60% pseudohyphae and 40% blastoconidia for the yeast [31]. Minimal inhibitory concentrations of NCT against conidia of *Aspergillus fumigatus*, *Aspergillus flavus*, and *Aspergillus niger* ranged between 16 and 65 µM NCT [33].

A more detailed insight in the activity against *Candida* species was provided by another study. Viable counts of *C. albicans*, *C. krusei*, *C. dubliniensis*, and *C. tropicalis* were reduced significantly by 1–3 log_10_ within 1–2 h at pH 7 and 37 °C. *Candida glabrata* was the most resistant species with a 2 log_10_ reduction after 4 h (4 log_10_ after 5 h) [32]. As expected, the killing curves declined slower at 20 °C [32].

Both hyphae and conidia of *Scedosporium apiospermum*, *Scedosporium boydii*, and *Lomentospora prolificans* (formerly *Scedosporium prolificans*) were killed by 55 mM (1.0%) NCT at pH 7.1 and 37 °C with a log_10_ reduction in cfu of 1–4 after 4 h and of 4 to >6 after 24 h [61]. LIVE/DEAD staining of conidia treated with 1.0% NCT for 0.5–3 h disclosed increased permeability of the cell wall and membrane.

These studies demonstrated the expected broad-spectrum fungicidal activity of NCT against different molds and yeasts, which is typical for active halogen compounds. Compared to other antiseptics, the killing by NCT is slow in buffer solution. However, the situation turns around in the presence of organic material.

### Fungicidal Activity in the Presence of Organic Material

Particularly proteins, peptides, and amino acids reduce the oxidation capacity of active chlorine compounds [23]. This occurs by reaction of the oxidizing chlorine with mainly thiols and thioethers, a so-called chlorine consumption effect [62]. However, a part of the active chlorine is transferred to amino groups (transchlorination, transhalogenation), resulting in formation of corresponding chloramines in equilibrium [63]. Transchlorination does not lead to a loss of oxidation capacity. The higher the reactivity of the active chlorine substance, the higher is the fraction that is reduced by proteinaceous material [62, 64]. For NCT—as one of the least reactive active chlorine compounds—this fraction is significantly lower [62]. Moreover, it transchlorinates at a significant amount with high and low molecular weight amines [22, 49]. Some of the formed corresponding low molecular weight chloramines exert higher microbicidal activity than NCT so that its net killing effect is enhanced in the presence of organic material as it occurs in the human body [27, 49]. This is very specific for NCT since all antiseptics up to date used in human medicine are impaired in their activity by organic matter.

Particularly, monochloramine (NH_2_Cl) formed from NCT plus ammonium ions (NH_4_^+^) plays a major role for this enhancement effect as shown already in the 1990s against some bacteria including mycobacteria and yeasts [27, 28]. This was confirmed against *A. flavus*, *A. fumigatus*, *C. albicans*, *Candida parapsilosis*, *Alternaria alternata*, *Fusarium moniliforme*, and *Penicillium commune*, which were killed by 1% (55 mM) NCT plus 0.005% (1 mM) NH_4_Cl at pH 7.4 and 37 °C within 10–30 min (<10 min by 1% NCT + 0.1% NH_4_Cl) [31, 33]. *Aspergillus flavus*, *A. fumigatus*, and *A. niger* were killed by 75–150 µM (0.0014–0.0027%) NCT in 5% fetal calf serum within 24 h [33]. In samples of nasal secretion, killing by plain 1% NCT was significantly hastened (30 min compared to ≥4 h), which is explained by the formation of monochloramine by halogenation of ammonium, too, which was found at a concentration of 1 mM in these samples. Monochloramine is more lipophilic than NCT and penetrates microorganisms easily, which explains its rapid microbicidal activity [19, 65].

Since inhaled NCT appears to be well tolerated, acute and chronic lung diseases, e.g., chronic obstructive pulmonary disease (COPD) and cystic fibrosis (CF), may become indications of interest. Therefore, we tested its bactericidal and fungicidal activity in artificial sputum medium (ASM; artificial CF-medium containing egg yolk emulsion, mucin type II from porcine stomach, salmon sperm-DNA, amino acids, diethylenetriamine penta-acetic acid, sodium chloride and potassium chloride [66]), mimicking the composition of cystic fibrosis mucus [67].

The medium was inoculated with bacteria (*S. aureus* including some MRSA strains, *P. aeruginosa*, *Escherichia coli*) or spores of fungi (*A. fumigatus*, *Aspergillus terreus*, *C. albicans*, *S. apiospermum*, *S. boydii*, *L. prolificans*, *Scedosporium aurantiacum*, *Scedosporium minutisporum*, *Exophiala dermatitidis*, *Geotrichum candidum*), and NCT was added at 37 °C. At a concentration of 1% (55 mM) NCT, bacteria and spores (10^7^–10^8^ cfu/ml each) were killed within 10 and 15 min to the detection limit of 10^2^ cfu/ml (reduction by 5–6 log_10_) [67]. A reduction by 2 log_10_ was still achieved by 0.1 and 0.3% NCT for bacteria and fungi, respectively, largely within 10–30 min. Measurements by means of iodometric titration showed oxidizing activity over 1, 30, 60, and >60 min at a concentration of 0.1, 0.3, 0.5, and 1.0% NCT, respectively, which matched the killing tests [67].

NCT demonstrated broad-spectrum microbicidal activity in the milieu of CF mucus at concentrations ideal for clinical use. Microbicidal activity of NCT in ASM was even stronger than in buffer solution, particularly pronounced with fungi [67]. This can clearly be seen in Table 1, where the integral method was used to reliably transform killing curves into one value [68]. The higher the value, the higher the microbicidal activity.

**Table 1:** Fungicidal activity (BA) of 1% NCT in ASM and phosphate buffer at pH 7 and 37 °C. Reproduced from: Gruber et al. [67]

Species	BA mean value (SD) in ASM	BA mean value (SD) in PO_4_
*C. albicans* ATCC 90028	0.71 (0.04)	0.0177 (0.0018)
*A. fumigatus* ATCC 204305	0.50 (0.03)	0.0087 (0.0020)
*A. terreus* ATCC 3633	0.51 (0.03)	0.0087 (0.0010)
*S. apiospermum* IHEM 21170	0.45 (0.04)	0.0031 (0.0002)
*S. boydii* isolate M36	0.52 (0.03)	0.0046 (0.0004)
*L. prolificans* IHEM 21176	0.42 (0.03)	0.0023 (0.0001)
*S. aurantiacum* V041-53	0.55 (0.04)	n.d.
*S. minutisporum* IHEM 21148	0.39 (0.02)	n.d.
*E. dermatitidis* CBS 207.35	0.42 (0.02)	n.d.
*G*. *candidum* isolate 267	0.37 (0.02)	0.0055 (0.0008)

*ASM* artificial sputum medium, *n.d.* no data available

### Postantifungal Effect and Attenuation of Virulence

A postantibiotic effect connected with loss of virulence of bacteria was found after a sublethal incubation time of only 1 min in 1% NCT in vitro and in vivo in a mouse peritonitis model [42] and with respective longer incubation times at lower concentrations of NCT [29]. A concentration- and time-dependent postantifungal effect was first demonstrated against *Candida* species with a lag of regrowth of up to 2.7 h [32]. Secreted aspartyl proteinases of *C. albicans* and *C. dubliniensis*, important virulence factors for these yeast species, decreased even before growth of the fungus was inhibited [32].

Pre-incubation of *Scedosporium* and *Lomentospora* in 1.0% NCT for 10–60 min delayed the time to germination of conidia by 2 to >12 h and reduced their germination rate by 10.0–100.0% [61]. Larvae of *Galleria mellonella* infected with 1.0 × 10^7^ conidia of *S. apiospermum* and *S. boydii* died at a rate of 90.0–100% after 8–12 days. The mortality rate was reduced to 20–50.0% if conidia were pre-incubated in 1.0% NCT for 0.5 h or if heat-inactivated conidia were used [61].

NCT and its dimethylated derivatives were shown to oxidize and inactivate virulence factors of bacteria. This was true for all tested toxins, i.e., shigatoxin of enterohaemorrhagic *E. coli* [44], and toxic shock syndrome toxin 1, enterotoxin A, and enterotoxin B, exfoliative toxin A, clumping factor, protein A of *S. aureus* [45]. Besides the mentioned impact on secreted aspartyl proteinases of *Candida* [32], up to date one further virulence factor of fungi was shown to be destroyed by NCT, namely gliotoxin of *A. fumigatus* [33]. Transferred to clinical application of NCT, this may abolish the ability of these fungi to invade tissue [32, 33].

### Considerations on Future Possibilities of Antifungal Therapy with *N*-Chlorotaurine

Clinical applications of NCT to treat fungal infections are rare so far. One case report regarding otomycosis has been reported, showing the potential of NCT [69]. A female patient suffering from external otitis was treated for 3 months with a combination of neomycin, polymyxin B, and hydrocortisone without success. Since 3 days she received also topical antimycotics, before she was admitted to the University hospital. After respective informed consent, the patient was treated topically with a cotton strip soaked with 1% NCT plus 0.1% dexamethasone to combine antimicrobial and deswelling drugs [30, 69]. The strip was changed once daily. Significant improvement of the symptoms was seen after 2 days, and she was dismissed from the hospital after 4 days. However, further 4 days later without treatment, she suffered from a relapse. At that time, fungal hyphae were clearly visible upon otoscopy, but no material was sent for fungal cultivation and identification. Topical treatment was started again with a 1% clotrimazole strip. Since neither the symptoms nor the diagnostic findings improved, treatment was changed again to 1% NCT plus 0.1% dexamethasone. Similar to the first cycle of application, the symptoms were reversed within 2 days. The objective findings of inflammation improved, too, but inflammation was not fully cleared. After 8 days of treatment, a small perforation of the tympanic membrane was found with an inflexible otoscope, and exudate was rinsing through the perforation. Therefore, in addition the middle ear was carefully rinsed daily with NCT plus dexamethasone via a small cannula introduced through the perforation. This kind of therapy was justified because of previous safety studies of NCT in the ear [70, 71]. A few days later, the inflammation was completely resolved, and the perforation disappeared after 13 days [69].

Further two unpublished applications in single patients against fungal infections on the skin were performed and successful. One suffered from several lesions on his arms (R. Arnitz, M. Nagl), and the second one from one large lesion on his right buttocks (B. Panhofer, M. Nagl). Due to the improved fungicidal activity (see above, [31, 61, 65]), in both cases 1% NCT plus 1% ammonium chloride was applied via soaked gauze two times daily. In both patients, the lesions could be cured within one week of treatment. No material for fungal cultures was sent before the beginning of therapy so that the fungus was confirmed only by fungal hyphae visible in microscopy of potassium hydroxide preparations.

In another patient, suffering from keratitis caused by *Fusarium* sp., and unsuccessfully treated with voriconazole eye drops, under 0.1% NCT plus 0.1% ammonium chloride eye drops the fungal cultures became negative and the inflammation disappeared (F. Pickl, M. Nagl). To avoid eye irritation, the concentration of NCT plus ammonium chloride has to be reduced to 0.1% [57], while 1% plain NCT is tolerated in the eye [56].

So far, all clinical cases and clinical studies suggest that NCT is a promising antiseptic agent for the topical treatment of fungal infections. Its broad-spectrum antifungal activity makes it applicable for various fungal species. Due to the outstanding tolerability, NCT can be applied to different body regions, including sensitive ones. In fungal infections with low exudation, e.g., eye and skin infections, the combination of NCT with ammonium chloride seems to be superior because of formation and rapid fungicidal activity of monochloramine (see above, [31, 61, 65]). If there is marked exudation, monochloramine is formed anyway at the site of application of NCT, and no addition of ammonium chloride is necessary. The disadvantage of the combination is its low stability for maximum three weeks even when stored at 4 °C so that it has to be mixed relatively promptly before use. NCT solution, by contrast, can be stored at room temperature for 3 weeks and at 4 °C for 1 year.

### Inhalation of *N*-Chlorotaurine

A special field of application of NCT is inhalation for treatment of various infections, including fungal ones. Bronchopulmonary infections are among the most frequent ones. While they usually can be overcome rapidly in young and immunocompetent patients, treatment of patients with risk factors is problematic, e.g., COPD, immunosuppression, and CF. Resistance against antibiotics accompanies chronic lung infections, and fungal infections in immunosuppressed people are life-threatening. Sometimes, also infections in young and healthy persons can be severe, e.g., influenza. In some indications, inhalation therapy with antibiotics is performed since the location of the infection can be reached by high concentrations (for instance, against *Pneumocystis jirovecii* and in CF) [72]. Inhalative antibiotics are licensed so far only in CF for reduction of lung function decline in patients with chronic pseudomonas infection [73]. According to a recent meta-analysis, there was no statistically significant difference in the efficacy for treatment of chronic *P. aeruginosa* lung infection in patients with CF between the approved inhaled drugs tobramycin, colistimethate sodium, aztreonam, and levofloxacin [74].

The good tolerability of NCT renders an antiseptic interesting for inhalation for the first time. In case of efficacy, such treatment could be a significant progress, for instance, against viral infections and multi-resistant pathogens, in CF or immunosuppressed patients. Moreover, prophylactic use in special indications, such as high-dose immunosuppression, is conceivable.

The following facts and previous studies suggest inhalation application of NCT.

The substance is also produced in the body by activated leukocytes [3, 5]. One function of this process is that the strongly toxic hypochlorous acid (HOCl) is detoxified by its reaction with taurine to form NCT (HOCl + taurine → NCT + H_2_O) [5, 9, 10]. This has been shown for lung epithelial cells, too [75]. The ciliary beat frequency of epithelial cells of the nasal mucosa, a very sensitive parameter for toxicity, was decreased only moderately and reversibly by 1% NCT, while an anesthetic solution used in the daily routine in otorhinolaryngology caused a severe and irreversible decrease in vitro [76].

Recently, we performed two studies on the tolerability of inhaled NCT in anesthetized pigs, whose lung is similar to the human one [77, 78]. In both studies, the animals inhaled 1% NCT (5 ml) versus 0.9% NaCl in a blind manner hourly, in total four times, on 1 day via the tracheal tube, which was connected with a nebulizer. In the second study, artificial inflammation was induced with *S. pyogenes* before the first inhalation. In both studies, there was no difference between 1% NCT and 0.9% saline in all tested parameters, which comprised oxygenation (e.g., arterial pressure of oxygen) and hemodynamics (e.g., pulmonary artery pressure). There were no toxic signs in histology and electron microscopy, and the function of the surfactant was not impaired. Systemic absorption of NCT was not detectable. A fivefold higher concentration of NCT (5%) showed a minimally elevated pulmonary artery pressure but no further differences to saline. Only by addition of 1% ammonium chloride to 1% NCT, which leads to formation of high amounts of monochloramine, some changes of parameters were seen, indicating the sensitivity of the model.

To test the tolerability of inhaled NCT over a longer period, we used a mouse inhalation model [79]. Mice (C57BL/6N, 8 week old) were put into a small chamber, which was connected to a nebulizer. NCT in aqueous solution (1%, 1 ml) versus 0.9% NaCl was nebulised for 5 min to the mice in the chamber once or twice daily over a period of 5 days in a first study and over 15 days in a second one. There was no difference between NCT and saline in all evaluated parameters, i.e., behavior, food, and water uptake, weight increase, blood count, and histology of the lungs.

To gain valuable hints for tolerability of NCT in humans, first pilot tests in humans, with three of the involved scientists (R. Arnitz, M. Nagl, B. Baumgartner), were performed under controlled conditions with reports to the respective health authorities. According to the study protocol, all inhaled 10 ml of 1% NCT over 10 min once and one person two times. When the subjects performed intensive breathing at the beginning, they felt a slight tickling in the throat, which led to occasional coughing. In the following, a non-disturbed normal inhalation was done over the whole period of 10 min. The only further sensation was a little chlorine taste in the mouth for some minutes after the end of the inhalation. Oxygenation was not impaired over the whole period, and there was no difference in the lung function measured by full body plethysmography before and after inhalation, including the forced expiratory volume in the first second (FEV_1_) and the airway resistance (*R*_eff_).

To gain further data including pharmacokinetics in man, one scientist performed a second according pilot test (M. Nagl). Briefly, the test person inhaled 1% NCT over 10 min as above, but once daily on 5 following days. Immediately after each inhalation, the oxidation capacity of sputum after addition of potassium iodide was determined by spectrophotometry [29]. Significant micromolar concentrations were detected 30 s and 1 min after the end of inhalation, while the oxidation activity came to the detection limit after 20 min at the latest. This result is in very good accordance with the absence of oxidative activity in bronchial lavages in pigs [77] and mice [79] subsequent to inhalations and indicates a very short half-life of inhaled 1% NCT. No systemic absorption could be measured. Notably, no additional adverse reactions to those found in the first pilot study (slight tickling in the throat, little chlorine taste) occurred during the repeated inhalations, and no adverse events were recognized in the periods between inhalations. Because of these encouraging results, a phase I study was initiated to prove the tolerability of inhaled NCT.

### Phase I Clinical Study on Inhalation of NCT

In this study, the tolerability of inhaled NCT was investigated for the first time in humans in a phase I clinical study. The study was performed double-blind and randomized with a parallel test group (1% NCT) and control group (0.9% NaCl as placebo). Two Austrian centers were involved, the hospitals Natters belonging to the Innsbruck University Hospital (M. Stein, H. Jamnig, P. Bauer) and Vöcklabruck in Upper Austria (R. Arnitz, B. Baumgartner). The study was performed in healthy, full age volunteers. In total, 24 subjects were tested, 12 were treated with NCT, and 12 with placebo. In each center, 6 subjects of the test and 6 ones of the control group were treated. The study medication consisted of 1% NCT in aqueous solution, the control solution of 0.9% NaCl. The single filling dose was 3 ml, which was inhaled over a period of up to 10 min using the AKITA JET inhalation system. The inhalation system delivered an amount of 1.2 ml to the mouthpiece and approximately 1 ml to the lungs of the subjects. One inhalation was performed on every day on 5 consecutive days.

The clinical phase of this study is finished in the meantime, and the detailed evaluation is ongoing. The primary criterion of evaluation, the forced expiratory volume in the first second (FEV_1_), was not reduced compared to the baseline and compared to 0.9% NaCl. Secondary criteria, such as subjective sensations, further lung function parameters, and blood analyses showed no abnormalities on a first view.

## Limitations

As an oxidizing antiseptic, NCT can be used only topically against microorganisms in vivo. Infections at locations inaccessible for irrigations cannot be treated with this substance.

Despite encouraging case applications, the efficacy of NCT against fungal infections remains to be proven in controlled, randomised clinical studies. The same is true for inhalation of NCT.

The fungicidal activity of NCT is lower than its bactericidal one. However, it is enhanced markedly by body fluids and exudate, which is uncommon for an antiseptic and explainable by its chemistry and transchlorination properties. In fungal infections with low exudate, addition of ammonium chloride may be considered to provide a rapid fungicidal action.

For storage exceeding three weeks, the 1% solution of NCT must be kept at 2–4 °C for 1 year with a negligible loss of 10% of its activity. The crystalline sodium salt shows a similar decay, but can be stored at minus 18 °C for 2 years or at lower temperatures for several years.

## Conclusion and Outlook

Because of its broad-spectrum microbicidal activity without development of resistance, endogenous occurrence, mild but sufficient activity, inactivation of toxins, local enhancement of microbicidal effects by exudate, and very good tolerability with no systemic distribution, NCT is a promising antiseptic that can be used topically in many body regions, including sensitive ones. Regarding inhalation, we aim to initiate phase II controlled, randomized, double-blind clinical studies in cystic fibrosis and chronic obstructive pulmonary disease.
